# B–NHL Cases in a Tertiary Pediatric Hematology—Oncology Department: A 20-Year Retrospective Cohort Study

**DOI:** 10.3390/life14050633

**Published:** 2024-05-16

**Authors:** Ioannis Kyriakidis, Iordanis Pelagiadis, Maria Stratigaki, Nikolaos Katzilakis, Eftichia Stiakaki

**Affiliations:** Department of Pediatric Hematology-Oncology & Autologous Hematopoietic Stem Cell Transplantation Unit, University Hospital of Heraklion & Laboratory of Blood Diseases and Childhood Cancer Biology, School of Medicine, University of Crete, 71003 Heraklion, Greece; kyriakidis@med.uoc.gr (I.K.); ipelagiadis@icloud.com (I.P.); mstratigaki@hotmail.com (M.S.); katzilaher@yahoo.gr (N.K.)

**Keywords:** Non-Hodgkin lymphoma, Burkitt lymphoma, children, adolescents, rituximab, immune reconstitution, drug toxicity, second neoplasm, biomarkers, positron emission tomography computed tomography

## Abstract

Non-Hodgkin lymphoma (NHL) is among the five most common pediatric cancer diagnoses in children and adolescents and consists of a heterogeneous group of lymphoid tissue malignancies –with B-cell-derived NHL accounting for nearly 80% of cases. Novel and high-throughput diagnostic tools have significantly increased our understanding of B-NHL biology and molecular pathogenesis, leading to new NHL classifications and treatment options. This retrospective cohort study investigated 17 cases of both mature B-cell NHL (Burkitt lymphoma or BL; Diffuse large B-cell lymphoma or DLBCL; Primary mediastinal large B-cell lymphoma or PMBCL; Follicular lymphoma or FL) and immature B-cell progenitor NHL (B-lymphoblastic lymphoma or BLL) that were treated in a tertiary Pediatric Hematology-Oncology Department during the last 20 years. Modern NHL protocols for children, adolescents, and young adults, along with the addition of rituximab, are safe and efficient (100% overall survival; one relapse). Elevated ESR was more prevalent than elevated LDH. Analyses have focused on immune reconstitution (grade ≥3 infections, lymphocyte and immunoglobulin levels recovery) and body-mass-index changes post-treatment, late effects (in 53% of patients), and the presence of histology markers BCL2, BCL6, CD30, cMYC, and Ki-67%. One patient was diagnosed with a second malignant neoplasm (papillary thyroid cancer).

## 1. Introduction

Non-Hodgkin lymphomas (NHLs) account for nearly 7% of all pediatric neoplasms [1]. Unlike Hodgkin lymphoma, which spreads contiguously, NHL is a systemic illness. In the same context, adults present predominantly with low-grade and clinically indolent NHL subtypes, while most pediatric NHL cases are high-grade and follow an aggressive clinical course. NHL is a diverse group of lymphoid tissue malignancies originating from B- or T-cells [2]. The current retrospective cohort study will emphasize only B-NHL cases, including both mature B-cell NHLs (Burkitt lymphoma or BL; Diffuse large B-cell lymphoma or DLBCL; Primary mediastinal large B-cell lymphoma or PMBCL; Follicular lymphoma or FL) and immature B-cell progenitor NHLs (B-lymphoblastic lymphoma or BLL). The clinical course and biological behavior of B-NHLs in childhood are highly variable, and each entity should be reviewed independently. The revised international pediatric NHL staging system (IPNHLSS) incorporates new histologic entities, along with other advancements, and facilitates reproducible data [3]. Recent discoveries in molecular platform analyses have driven a rapid transition from a primarily microscopic to a molecular classification [4]. The identification of novel genetic drivers that confer distinct clinical, phenotypic, and prognostic features has recently led to the fifth edition of the World Health Organization (WHO) Classification of Haematolymphoid Tumors [5]. Most children and adolescents with B-NHL and access to modern regimens display excellent outcomes despite the short- and long-term treatment-related toxicities. However, outcomes in PMBCL and refractory or relapsed disease remain problematic [6,7].

This study aims to describe all B-NHL cases treated in a tertiary Pediatric Hematology-Oncology Department during the last 20 years, including late effects, anthropometric measures, and immune reconstitution.

## 2. Materials and Methods

A retrospective cohort study was conducted, and the data corresponded to 17 cases treated with B-NHL in a tertiary Pediatric Hematology-Oncology Department (children, adolescents, and young adults up to 22 years old). The diagnosis was based on medical history, physical examination, laboratory studies, imaging, and biopsies, while it was established by cytogenetic and histopathology studies, adhering to the respective guidelines [5,8]. Medical records were reviewed, and information on symptoms was extracted from medical notes. Besides classic B-cell markers, we have studied BCL2, BCL6, CD30, and cMYC presence in association with clinical traits and laboratory indices. Immunohistochemistry studies by subtype are listed in Supplementary Table S1. Fluorescence In Situ Hybridization (FISH) assays followed the histopathology findings as per current guidelines [5,8]. The body weight, height, and body mass index (BMI) were recorded both at diagnosis and one month after the end of treatment. The respective percentiles were obtained from the Child’s Health Booklet, which is issued by the Greek Ministry of Health (https://bit.ly/3GswxJJ; accessed on 24 April 2024) and follows the WHO growth charts (https://www.who.int/tools; Child Growth Standards and Growth reference data for 5–19 years; accessed on 24 April 2024). Statistical analysis was performed using IBM SPSS Statistics version 29.0.1.0 (IBM Corp., Armonk, NY, USA). A priori analysis, conducted using G*Power 3.1.9.2 software (Heinrich-Heine-Universität, Düsseldorf, Germany), indicated that the current sample size was sufficient to achieve considerable actual power. The Shapiro–Wilk test of normality was performed to identify normally distributed variables. Non-parametric tests were used in data that did not follow a normal distribution (Mann–Whitney U, Kruskal–Wallis, chi-square tests, and Spearman’s rank correlation coefficient r_S_). The significance level was set at 0.05. The swimmer plot was designed in RStudio version 2023.09.1 + 494 (Posit Software, PBC, Boston, MA, USA). The data correspond to the period from 1 May 2004 to 30 April 2024.

## 3. Results

*BL predominance, advanced stage, and diagnostic delay*. Our tertiary Pediatric Hematology-Oncology Department fulfills the respective needs of almost 100,000 children in the region of Crete (total population of 617,360). So, the mean incidence of B-NHLs in Crete, Greece, is estimated at 8.5 cases per 1,000,000 children annually. Regarding BL alone, the incidence was estimated at 5 cases per 1,000,000 children annually. In concordance with previous reports, our cohort’s median age at diagnosis was 9.1 years (range: 4.3 to 22.3). B-NHL has a male predilection, and this is widely known. In our B-NHL cohort, males were 76.5% and females 23.5% (male-to-female ratio 3.3), while, interestingly, all BL cases were males [1]. The main epidemiologic traits of our cohort are summarized in Table 1. The same traits by subtype can be found in Supplementary Table S1. As expected, BL cases outnumbered all other B-NHL types. Pediatric-type FL is overrepresented in the current cohort due to its limited size. In terms of staging, more than half of the patients were diagnosed with stage III disease. Regarding risk stratification, most cases corresponded to strata B and R2 according to the French Society of Pediatric Oncology (FAB/LMB) and Berlin-Frankfurt-Münster (BFM) protocols, respectively. The median time interval between the onset of symptoms and the confirmed diagnosis of B-NHL (i.e., the diagnostic delay) was 30 days, but that period was highly variable between cases (2 to 120 days). Interestingly, the diagnosis establishment lasted longer than two months in 1 out of 4 cases. Due to its aggressive B-cell features, BL was diagnosed earlier than other B-NHLs (median 19 days; range: 2 to 84 days). However, no statistically significant differences were detected among B-NHL groups. Markedly, delayed diagnosis was not associated with response to treatment, relapse, or outcome. In most cases, B-NHL manifested in the abdomen (41%; all were BL cases), followed by the mediastinum and the neck (12% each). Other uncommon locations should not be ruled out while investigating for B-NHL (such as the orbit, the sinuses, the musculoskeletal system, etc.). The diagnosis delay cannot be attributed to rare B-NHL locations or the type of B-NHL (*p* > 0.05), but adolescence and young adulthood were associated with significantly longer periods to reach diagnosis (r_S_ = 0.583; *p* = 0.023). The median age at diagnosis was significantly lower for BL and BLL and higher for all other B-NHL types (*p* = 0.046).

*Rare sites of involvement correlate with a higher disease stage*. The symptoms ranged from mild to severe and life-threatening (such as superior vena cava syndrome in one patient and acute kidney injury in another). Fatigue was the most common symptom (47%), followed by painless swelling (29%). B-symptoms were exhibited in only two patients (5.9%). Other symptoms affecting >10% of patients were vomiting (29%), abdominal pain, and mild fever (24% each), all referring to patients with BL. Rare sites of involvement were linked with higher stages of disease (*p* = 0.006), and the Deauville score after two courses of treatment was higher among DLBCL and PMBCL patients (*p* = 0.045).

*A high white blood cell (WBC) count at diagnosis is associated with disease aggressiveness but not with B-NHL type or prognosis*. Routine laboratory work-up at diagnosis (Table 1) has little to offer unless the disease has affected the bone marrow. Only two BL cases were found with a WBC count outside the normal range (12%; 20% of BL). WBC at diagnosis was not significantly different between B-NHL types. Strikingly, the WBC did not associate with any other parameters in our analysis other than with Ki-67 % (r_S_ = 0.626; *p* = 0.017) and cMYC positivity in histology investigations (14,050 vs. 7300 for negative specimens; *p* = 0.04). Unlike WBC, lymphocyte count at diagnosis was not associated with any categorical variable in this study. The lymphocytic population at diagnosis has been linked with fewer days from the onset of symptoms to diagnosis (r_S_ = −0.537; *p* = 0.039). Post-hoc analysis revealed significantly higher relative mononuclear cell population (%mono) at diagnosis in higher stages of B-NHL (≥III; *p* = 0.013). Intriguingly, patients who did not respond to initial chemotherapy regimens had a higher %mono at diagnosis than good responders (18% vs. 8.9%; *p* = 0.02). Moreover, patients diagnosed with B-NHL at common sites (abdomen, mediastinum, and neck) had higher %mono at diagnosis than those with rare locations (11.4% vs. 5.7%; *p* = 0.004). PLTs at diagnosis were positively correlated with Ki-67% (r_S_ = 0.599; *p* = 0.024). The platelet-lymphocyte ratio in this cohort was correlated with ESR (r_S_ = 0.526; *p* = 0.044), while the lymphocyte-monocyte ratio was higher in stage IV disease (*p* = 0.04). As regards FAB/LMB risk stratification, individuals in stratum C had significantly lower Hb than those in stratum B (9.6 vs. 12 mg/dL; *p* = 0.015), which was not found for BFM risk strata.

*Elevated ESR is more prevalent than elevated LDH*. Blood chemistry investigations at diagnosis are described in Table 2, and their reference values are available in Supplementary Table S2. High ESR was a consistent finding, followed by LDH. Besides WBC and PLT at diagnosis, Ki-67 has also been correlated with CRP (r_S_ = 0.679; *p* = 0.015). LDH levels at diagnosis did not differ among B-NHL types or stages. Additionally, significantly higher AST and lower albumin values at diagnosis were observed in higher strata of BFM risk stratification (*p* = 0.023 and *p* = 0.013, respectively). High serum LDH and AST and low albumin at presentation were significantly associated with a shorter period to establish B-NHL diagnosis (r_S_ = −0.517 with *p* = 0.048; r_S_ = −0.596 with *p* = 0.019; and r_S_ = 0.575 with *p* = 0.025, respectively).

*Immune reconstitution after B-NHL treatment*. Table 3 presents the levels of immunoglobulins (Ig) and IgG subclasses initially and at the end of chemotherapy, along with the period needed to recover at normal-for-age levels. The median duration required for lymphocytes to exceed 1000/μL after the end of treatment was more than a month (34.5 days; range: 0 to 487). The highest recovery periods were recorded with PMBCL and DLBCL (127 and 149 days, respectively), but no significant associations were calculated (21 days in BL; range: 2 to 487). In terms of treatment protocols, more extended periods to lymphocyte recovery were registered with the DA-EPOCH-R regimen, but no statistically significant associations were noted. Strikingly, rituximab administration was not associated with longer periods of lymphopenia post-treatment. ESR and platelet-to-lymphocyte ratio at diagnosis correlated strongly and positively with the lymphopenia period after the end of chemotherapy (r_S_ = 0.811; *p* < 0.001; and r_S_ = 0.534 with *p* = 0.049, respectively). Furthermore, hypoalbuminemia at diagnosis was linked with long-lasting lymphocyte recovery to counts above 1000/μL post-therapy (r_S_ = −0.591; *p* = 0.026). Levels of Igs at diagnosis were highly variable: (i) normal-for-age IgA in 58% of the cohort -one patient only with low levels, one patient with high levels, and three patients with upper limit levels; (ii) normal-for-age IgM in 83% -one patient with low and one with upper limit levels; (iii) normal-for-age IgG in 58% -two patients with low and three patients with high levels. At the end of therapy, low-for-age IgM was ubiquitous (71.4%), while low IgA and IgG were measured only in one out of five cohort patients. High AST and LDH at diagnosis correlated strongly and negatively with IgA levels (r_S_ = −0.797 with *p* = 0.002, and r_S_ = −0.594 with *p* = 0.042, respectively). Remarkably, patients with positive Epstein–Barr virus (EBV) IgG titers tended to have lower total IgG levels at diagnosis (871.5 vs. 1600 mg/dL; *p* = 0.028). IgG levels at diagnosis were significantly lower in strata of higher risk for both FAB/LMB and BFM risk stratification systems (r_S_ = −0.583 with *p* = 0.047 and r_S_ = −0.717 with *p* = 0.009, respectively). Inaugural high serum creatinine levels were linked with low pre-treatment IgM and IgG levels (r_S_ = −0.603 with *p* = 0.038 and r_S_ = −0.578 with *p* = 0.049, respectively). As anticipated, low serum IgM and IgG levels at the end of treatment required significantly more days to recover within normal-for-age levels (r_S_ = −0.769 with *p* = 0.003; and r_S_ = −0.619 with *p* = 0.032, respectively). Post-treatment, IgM and IgG levels were lower among patients administered rituximab, but this association did not reach significance levels. Apparently, the patient who relapsed had significantly higher recovery periods for IgA, IgM, and IgG. An interesting finding was that recovery of serum IgA to normal-for-age levels lasted significantly more days in patients with a high relative mononuclear cell population at diagnosis (r_S_ = 0.763; *p* = 0.004), while IgG recovery lasted longer in patients with high cystatin-C at diagnosis (r_S_ = −0.809; *p* = 0.028). Contrarily, IgM recovery was not associated with any of the studied parameters. Grade ≥ 3 infection development was only weakly associated with low IgA levels at diagnosis (one-sided *p* = 0.034).

*B-NHL survivors are prone to obesity*. Tall-for-age patients (≥90th percentile) at diagnosis corresponded to one-third of the cohort (5/15 vs. none with low height ≤10th percentile; two-sided *p* = 0.009). Also, patients with height ≥2 SDs at diagnosis were more likely to belong to BFM risk stratum 4 (*p* = 0.02). Elevated body weight (≥90th percentile) at diagnosis was documented in 3/15 patients (20%), and low body weight was recorded in only one patient (≤10th; NS), while no deviations for BMI were observed at diagnosis. All but one patient had greater BMI values at follow-up one month after the end of their treatment than those recorded at diagnosis (22.3 ± 4.5; *p* < 0.001), and 25% of the cohort were obese post-treatment, highlighting the importance of metabolic disorders and dietary guidance for cancer survivors. Lack of response to chemotherapy has been correlated with a higher BMI percentile post-treatment (*p* = 0.017 for each). The mean change in BMI between the time of diagnosis and one month after the end of treatment was +19.4%.

*Late effects are prevalent*. More than half of patients (53%) developed late effects that require regular medical attention or are associated with disability. Elevated serum GGT and creatinine at diagnosis were associated with their prevalence (*p* = 0.029 each). Three patients (18%) were diagnosed with hepatic focal nodular hyperplasia (FNH; two males and one female). Endocrine late effects were observed in three patients (18%) and were defective thyroid function, thyroid carcinoma, and pathologic fractures, respectively. After their treatment, three more children experienced cardiovascular events and conditions (18%): two cases of hypertension and one of internal jugular vein thrombosis (with underlying *MTHFR* C677T homozygosity). Cardiovascular effects were more likely to occur in stratum C of the FAB/LMB risk stratification system (Fisher’s exact test; *p* = 0.029). Three children were diagnosed with more than one late effect, including one with dysplastic nevi, one with encephalopathy due to thiamine deficiency, and one with a psychiatric disorder.

**Figure 1:**
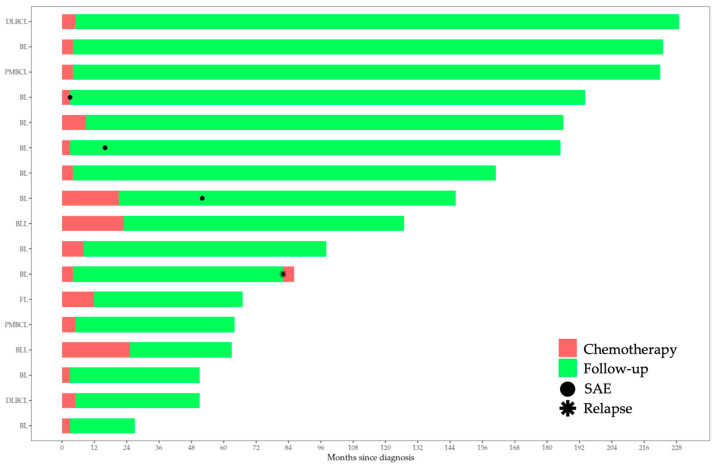
Swimmer plot of the B-NHL cohort.

*Modern BFM protocols are safe and effective*. Regarding treatment protocols, the vast majority of this cohort was treated adhering to BFM regimens (80%; 47% with B-NHL BFM 2004), while 40% was administered with rituximab (introduced in 2011 for NHL in the Department). Only one patient (back in 2008) underwent radiotherapy. Regarding survival rates, all patients are alive to date. Poor responders to chemotherapy did not inevitably undergo bone marrow transplantation (BMT). In this cohort, one BL patient did not respond to the initial chemotherapy regimen, while another BL patient relapsed (83 months after his initial diagnosis). A swimmer plot including the serious adverse events (SAEs; grade ≥ 3) and the relapsed case was drawn (Figure 1). SAEs correspond to one case of thrombosis, one case of prolonged hypogammaglobulinemia (>1 year), and one case of thyroid cancer.

## 4. Discussion

This retrospective cohort study confirms our knowledge of B-NHL and BL incidence (8.5 and 5 cases per 1,000,000 person-years, respectively, increasing incidence with age). Beyond the 17 B-NHL cases described herein, three more children were treated in our department for T-NHL during the corresponding period, so the mean incidence of NHL was determined at 10 cases per 1,000,000 per year. These rates are similar to previously reported incidence rates for NHL and BL in childhood and adolescence [6,9,10,11]. An annual percent increase of 1% has been documented for the decade 2000–2019 in NHL incidence, and this trend is confirmed by our data [11]. Incidence rates of NHL in Southern Europe seem to be higher than in other regions of the world, especially among adolescents, and with an increasing rate, especially for BL [12]. Nevertheless, five-year overall survival has climbed from 70% in the early 1990 s to nearly 90% in many developed countries, even by eliminating radiotherapy and chemotherapy alone in most protocols [13]. The male predominance in NHL and primarily in BL has been corroborated by our cohort results [1]. Staging in pediatric NHL remains an issue, especially in developing countries, affecting treatment selection and, of course, outcomes [14]. In Greece, there are two essential advantages to this issue: easy access to primary care and laboratory investigations and, recently, the accessibility to more sophisticated diagnostic tools (such as positron emission tomography, computed tomography, and comprehensive genetic testing). However, most patients were diagnosed with stage III and IV disease [15]. The median time interval between the onset of symptoms and diagnosis was high (30 days), but comparable with previous studies, and did not associate with residing more than 100 km away from our department, staging, or outcome. In agreement with our analysis, a relevant retrospective study from Korea reported a higher lag time for adolescents than children, which was principally attributed to patient delay. Adolescence is a developmental period with many psychological changes, including autonomy, omnipotence, and a tendency to take risks, along with a reluctance to disclose symptoms of their changing body, thus leading to unique health-seeking patterns [16]. Besides age, our study showed that lag time is significantly shorter in patients with a high lymphocyte count, elevated LDH, increased AST, and low albumin levels at diagnosis, implying that these indices might be of value for diagnosis. Early referral and timely supportive care are still significant global issues for improving survival rates [15,17]. A national cohort study from Sweden reported high fetal growth (≥2 SDs vs. 0 to <1 SD: HRadj = 1.64; 95% CI = 1.19 to 2.25; *p* = 0.002) as a risk factor for NHL, whereas a former indigenous study from Greece correlated NHL risk with high birth weight (odds ratio: 1.42; 95% CI: 1.04 to 1.92) [9,18]. Our cohort displayed high patient heights but no significant deviations in weight. A relevant meta-analysis showed null associations between birth weight and B-NHL [19].

Currently, two risk systems are used to stratify patients with mature B-cell NHL and tailor therapy. Bulk and B symptoms are not prognostic, unlike in HL. Elevated LDH at diagnosis is being used as a biomarker of dismal prognosis. However, its prognostic value is lower than in HL, and a higher cut-off is required to yield more definite associations (>2000 U/L) [20]. In our cohort, only 73% presented with elevated LDH, and its values did not predict adverse outcomes. Beyond LDH, low albumin has been associated with treatment-related mortality, but a robust prognostic biomarker in pediatric B-NHL is lacking [20,21]. The literature reports support the prognostic value of beta-2-microglobulin levels on NHL survival rates [22]. Our cohort displayed values within the normal range at diagnosis, whenever measured, maybe because none of these patients were deceased. The lymphocyte-to-monocyte ratio has also been suggested as a prognostic biomarker [23]. In the current cohort, a high lymphocyte-to-monocyte ratio was observed in stage IV disease; however, it did not preclude favorable outcomes. Of note, a significantly higher %mono at diagnosis was observed in higher stages of B-NHL and among patients who failed to respond to chemotherapy. Peripheral blood mononuclear cells in NHL patients play an essential role in disease aggressiveness and suppressed immune functions [24,25]. Moreover, serum ceruloplasmin and dehydroepiandrosterone sulfate (DHEA-S) levels at diagnosis in our cohort did not correlate with any of the studied parameters. Utilization of isobaric tags for relative and absolute quantification (iTRAQ)-based proteomic analysis combined with Ingenuity Pathway Analysis revealed that acute phase response signaling and liver X receptor/retinoid X receptor (LXR/RXR) activation play a pivotal role in NHL tumorigenesis. Based on the latter study, elevated serum S100A8 protein and leucine-rich alpha-2-glycoprotein 1 (LRG1) are promising candidate biomarkers for pediatric NHL, identifying 97% of patients [26]. Histology markers are crucial for diagnosis and selecting the proper treatment protocol. BCL2 positivity in histological investigations was universal in non-BL cases (*p* = 0.012), and CD30+ staining was bright explicitly among PMBCL cases and exclusive for BL (*p* = 0.046).

It has been long known that immune homeostasis is involved in B-NHL pathogenesis. Low pretreatment IgG levels were linked with inferior outcomes in NHL and have been proposed as a prognostic factor [23]. In our cohort, low IgG at diagnosis correlated with higher risk strata (along with previous EBV infection) but not with outcome. Diminished Ig levels at diagnosis combined with impaired renal function have been implicated in the emergence of severe infections in hematologic malignancies [27]. Our data substantiate this theory through a complex immunologic circuit: increased creatinine levels correlated with low IgM and IgG levels at diagnosis, low C3 levels and increased cystatin-C correlated with low platelet counts, and grade ≥ 3 infections associated with low initial IgA levels. Nevertheless, Ig levels at diagnosis were highly variable, with hyperimmunoglobulinemia being more prevalent than hypoimmunoglobulinemia, a fact that has been previously described [28,29].

Immune reconstitution following standard-dose chemotherapy regimens usually occurs within 3 to 6 months from the cessation of treatment and may be withheld either due to rituximab administration or BMT [30]. Rituximab treatment leads to rapid and profound depletion of pre-B, mature B-cells, and CD3 + CD20+ T-cells, but sparing plasma cells and B-cell precursors. This effect usually lasts 6–12 months, while Ig depletion lasts 5–12 months and may require substitution. The subsequent increase in immature and transitional B-cells leads to a reduced function of B-cells as antigen-presenting cells and a dysfunction of CD4+ T-cells, thus impairing cytotoxic T-cell-specific responses. CD20 elimination also affects Th17 cells, which results in less protected mucosal barriers and decreases pathogen clearance [31]. Despite these facts, and in keeping with our results, relevant meta-analyses have shown that adding rituximab to NHL chemotherapy regimens does not affect meaningful infection rates [7]. B-cell compartment reconstitution takes place 3–4 months after the end of treatment, and prolonged (>800 days) low levels of IgA, IgM, and IgG were reported in 25%, 13%, and 9% of the pediatric population with NHL that was treated with rituximab, respectively [32,33]. Our results confirm these data and show that Ig and lymphocyte recovery intervals did not differ significantly between rituximab recipients and non-recipients. In addition, lymphocyte and Ig classes’ recovery to normal-for-age levels lasted longer in cases with high-stage disease, acute kidney injury, high platelet-lymphocyte ratio, %mono, ESR, and low albumin levels at diagnosis. Despite the risk of prolonged hypogammaglobulinemia in children and adolescents treated for NHL, severe infections are rare [34]. In our cohort, 53% of patients developed a grade 3 infection or febrile neutropenia, but no one experienced a grade 4 or 5 infection.

Late effects are an emerging issue for children and adolescents treated for NHL. A recent BFM report calculated the cumulative incidence of second malignant neoplasms (SMN) at 5.3% at 20 years. Female sex, BLL diagnosis, and cancer-predisposing conditions have been identified as independent risk factors for the development of SMN. Carcinomas were more prevalent than acute myeloid leukemia and second lymphoid malignancies (cumulative incidence 1.9% vs. 0.7%, each) [35]. Likewise, the St. Jude Lifetime cohort study noted that 10-year survivors of NHL had a 6.3-fold increase in SMN rate (10.5%) compared with the expected rate of corresponding de novo cancers in the general population [36]. In our cohort, only one BL patient developed thyroid cancer 2.5 years after the initial diagnosis, while another developed non-melanoma skin cancer. According to growing evidence, prevention strategies aim to mitigate late toxicity through cumulative dose reductions and eliminating radiation [37].

## 5. Conclusions

This study has several limitations, with its retrospective nature and small cohort size being the major ones. However, it is widely recognized that rituximab revolutionized treatment in children and adolescents with B-NHL. Molecular advances and PET/CT scans have also reformed classification and staging, respectively, and new entities have emerged (such as high-grade/large B-cell lymphoma with 11q aberration). Nevertheless, with survival rates approaching 90%, more and more survivors of childhood NHL are expected to encounter chronic treatment-related health conditions. Our data confirm the efficacy and safety of modern NHL protocols for children and adolescents.

## Figures and Tables

**Table 1:** Characteristics of the B-NHL cohort.

	n = 17	Sex (M:F)	Median Age at Diagnosis (Range)
**B-NHL classification**			
BL	10 (58.8%)	10:0	7.7 (4.3–10.4)
PMBCL	2 (11.8%)	1:1	17.6 (12.8–22.3)
DLBCL	2 (11.8%)	1:1	12.5 (9.2–15.8)
BLL	2 (11.8%)	1:1	4.9 (4.7–5)
FL	1 (5.9%)	0:1	14.1
**Murphy staging**			
Stage I	6%		
Stage II	12%		
Stage III	53%		
Stage IV	29%		
**FAB/LMB risk stratification**			
A	-		
B	87%		
C	13%		
**BFM risk stratification**			
R1	-		
R2	53%		
R3	20%		
R4	27%		

F = female; M = male.

**Table 2:** Complete blood count and blood chemistry findings at diagnosis.

Work-Up	Median	Range	% of Cohort with Abnormal Values for Age
WBC (/μL)	8500	3800 to 62,500	12%
Lymphocytes (/μL)	2204	531 to 13,750	20%
Lymphocyte proportion ^†^ (%)	27.5 ± 13.3	20.2 to 34.9	12%
Relative neutrophil count ^†^ (%)	60.3 ± 15.2	51.8 to 68.7	18%
Relative mononuclear cell population ^†^ (%)	9.5 ± 4	7.3 to 11.7	41%
Percentage of eosinophils ^†^ (%)	2.3 ± 2	1.2 to 3.7	None
Hemoglobin ^†^ (g/dL)	11.6 ± 1.4	10.9 to 12.4	6%
Platelet count ^†^ (/μL)	330,060 ± 117,736	265,400 to 395,800	6%
Platelet-to-lymphocyte ratio	152.6	2.8 to 580	60%
Lymphocyte-to-monocyte ratio	3.5 ± 2.5	2.1 to 4.9	53%
LDH (U/L)	397	194 to 14,640	73%
ESR (mm/1 h)	26	10 to 110	93%
CRP (mg/dL)	0.67	0.03 to 5.5	54%
AST (U/L)	30	18 to 281	40%
ALT (U/L)	18	9 to 67	20%
GGT (U/L)	14	8 to 110	33%
Albumin ^†^ (g/dL)	3.9 ± 0.5	3.6 to 4.2	33%
Creatinine (mg/dL)	0.56	0.4 to 1.2	13%
C3 (mg/dL)	128.5	119 to 184	17%
Cystatin-C (mg/L)	0.62	0.54 to 1.04	0

C3 = complement component 3; CRP = C-reactive protein; AST = glutamic aspartate transaminase or SGOT; ALT = Alanine transaminase or SGPT; ^†^ normally distributed (Shapiro–Wilk test of normality; *p* > 0.05), therefore mean, standard deviation (SD) and 95% confidence intervals (CI) fit better for description.

**Table 3:** Immunoglobulins and immunoglobulin G subclasses.

Ig	Levels at Diagnosis	Levels at the End of Chemotherapy	Days to Recover at Normal Levels
IgA (mg/dL)	158.7 (±108.1)	106.5 (±59.5)	0 (0 to 3530)
IgM (mg/dL)	112.5 (±48.8)	39.6 (15.6 to 195)	359 (0 to 3530)
IgG (mg/dL)	1110.8 (±431.4)	766.7 (±257.8)	0 (0 to 1483)
IgG1 (mg/dL)	563.8 (±258)	620.7 (±298.9)	NA
IgG2 (mg/dL)	168.8 (±81.1)	270.3 (±129.4)	NA
IgG3 (mg/dL)	32.2 (±17.7)	34.1 (±21.9)	NA
IgG4 (mg/dL)	11.4 (±9.3)	19.1 (±14)	NA

Normally distributed values are described with mean ± SD, while values that did not follow normal distribution are described with median and range; NA = not available.

## Data Availability

The data used to support the findings of this study are available from the corresponding author upon request.

## Supplementary Materials

The following supporting information can be downloaded at: https://www.mdpi.com/article/10.3390/life14050633/s1, Table S1: Characteristics of the B-NHL cohort by subtype; Table S2: Reference values for blood chemistry work-up. Reference [38] is cited in the Supplementary Materials.
