# Supplementary material for: B–NHL Cases in a Tertiary Pediatric Hematology—Oncology Department: A 20-Year Retrospective Cohort Study
Source: Life (Basel). 2024 May 16;14(5):633. doi: 10.3390/life14050633 (PMC11122206; doi:10.3390/life14050633)
Supplement: Supplementary file 1 [file life-14-00633-s001.zip › life-3019938-supplementary.pdf]

Table S1. Characteristics of the B-NHL cohort by subtype.

	B-NHL cohort	BL	PMBCL	DLBCL	BLL	FL
Murphy staging						
Stage I	6%	-	-	50%	-	-
Stage II	12%	20%	-	-	-	-
Stage III	53%	60%	100%	-	-	100%
Stage IV	29%	20%	-	50%	100%	-
FAB/LMB risk stratification						
A	-	-	-	-	-	-
B	87%	80%	100%	100%	100%	100%
C	13%	20%	-	-	-	-
BFM risk stratification						
R1	-	-	-	-	-	-
R2	53%	50%	100%	-	50%	100%
R3	20%	10%	-	100%	50%	-
R4	27%	40%	-	-	-	-
Histology markers §						
BCL2+	27%	-	100%	100%	100%	-
BCL6+	82%	85%	100%	100%	-	100%
CD30+	25%	-	100%	-	-	-
cMYC+	65%	100%	-	50%	-	-
Median Ki-67%	95%	99%	85%	100%	85%	30%

§ List of other immunohistochemistry markers that have been utilized to establish the diagnosis: BOB1, CAM5.2, CD1a, CD3, CD4, CD5, CD10, CD15, CD19, CD20, CD21, CD22, CD23, CD25, CD27, CD34, CD35, CD38, CD43, CD45 (LCA), CD57, CD68, CD79a,

CD79b, CD99, CD103, CD117, CD123, CD138, CD200, cMYC, cyclinD1, DBA44, desmin, EBER1/2, EMA, EZH2, FMC7, HHV8, Ig, IRF4/MUM1, MNF116, MPO, myogenin, NSE, OCT2, p63, PAX5, S100, SMA, TCL1, TdT, vimentin; The relapsed BL case was CD10+, CD19+, CD20+, BCL6+ and cMYC+ (confirmed with FISH for *MYC*::*IGH*; Ki-67 100%); IRF4/MUM1 was positive only in one PMBCL and one DLBCL patient.

Table S2. Reference values for blood chemistry work-up presented in Table 2.

Work-up	Normal range
LDH	Birth to 14 days: 309 – 1222 U/L<br>15 days to <1 year: 163 – 452 U/L<br>1 to <10 years: 192 – 321 U/L
ESR	Children: 0 – 10 mm/hr<br>Female adolescents and young adults: 0 – 20 mm/hr<br>Male adolescents and young adults: 0 – 15 mm/hr
CRP	Birth to 14 days: 0.03 – 0.61 mg/dL<br>15 days to <15 years: 0.01 – 0.1 mg/dL<br>15 to <19 years: 0.01 – 0.17 mg/dL
AST	Birth to 14 days: 32 – 162 U/L<br>15 days to <1 year: 20 – 67 U/L<br>1 to <7 years: 21 – 44 U/L<br>7 to <12 years: 18 – 36 U/L<br>12 to <19 years (female): 13 – 26 U/L<br>12 to <19 years (male): 14 – 35 U/L
ALT	Birth to <1 year: 5 – 33 U/L<br>1 to < 13 years: 9 – 25 U/L

	13 to 19 years (female): 8 – 22 U/L<br>13 to 19 years (male): 9 – 24 U/L
GGT	Birth to 14 days: 23 – 219 U/L<br>15 days to <1 year: 8 – 127 U/L<br>1 to <11 years: 6 – 16 U/L<br>11 to <19 years: 7 – 21 U/L
Albumin	Birth to 14 days: 3.3 – 4.5 g/dL<br>15 days to <1 year: 2.8 – 4.7 g/dL<br>1 to <8 years: 3.8 – 4.7 g/dL<br>8 to <15 years: 4.1 – 4.8 g/dL<br>15 to <19 years (female): 4.0 – 4.9 g/dL<br>15 to <19 years (male): 4.1 – 5.1 g/dL
Creatinine	Birth to 14 days: 0.32 – 0.92 mg/dL<br>15 days to <2 years: 0.1 – 0.36 mg/dL<br>2 to <5 years: 0.2 – 0.43 mg/dL<br>5 to <12 years: 0.31 – 0.61 mg/dL<br>12 to <15 years: 0.45 – 0.81 mg/dL<br>15 to <19 years (female): 0.49 – 0.84 mg/dL<br>15 to <19 years (male): 0.62 – 1.08 mg/dL
C3	Cord blood (term): 57 – 116 mg/dL<br>1 month: 53 – 124 mg/dL<br>2 months: 59 – 149 mg/dL<br>3 months: 64 – 131 mg/dL<br>4 months: 62 – 175 mg/dL<br>5 months: 64 – 167 mg/dL<br>6 months: 74 – 171 mg/dL

<table>
<tr><td></td><td>7 to 9 months: 75 – 166 mg/dL
10 to 12 months: 73 – 180 mg/dL
1 year: 84 – 174 mg/dL
2 years: 81 – 170 mg/dL
3 years: 77 – 171 mg/dL
4 to 5 years: 86 – 166 mg/dL
6 to 8 years: 88 – 155 mg/dL
9 to 10 years: 89 – 195 mg/dL
Young adults: 83 – 177 mg/dL</td></tr>
<tr><td>Cystatin-C</td><td>Birth to <2 years (female): 0.682 – 1.218 mg/L
Birth to <2 years (male): 0.666 – 1.262 mg/L
2 to <11 years (female): 0.654 – 1.074 mg/L
2 to <11 years (male): 0.651 – 1.075 mg/L
11 to <15 years (female): 0.64 – 1.132 mg/L
11 to <15 years (male): 0.684 – 1.196 mg/L
15 to 18 years (female): 0.597 – 1.029 mg/L
15 to 18 years (male): 0.706 – 1.174 mg/L</td></tr>
<tr><td>Platelet-to-lymphocyte ratio</td><td rowspan="2">According to
Moosmann, J., Krusemark, A., Dittrich, S., Ammer, T., Rauh, M., Woelfle, J., Metzler, M., & Zierk, J. (2022). Age- and sex-specific pediatric reference intervals for neutrophil-to-lymphocyte ratio, lymphocyte-to-monocyte ratio, and platelet-to-lymphocyte ratio. International journal of laboratory hematology, 44(2), 296–301. https://doi.org/10.1111/ijlh.13768 [38]</td></tr>
<tr><td>Lymphocyte-to-monocyte ratio</td></tr>
</table>
